# Configurations for obtaining in-consultation assistance from supervisors in general practice training, and patient-related barriers to trainee help-seeking: a survey study

**DOI:** 10.1186/s12909-020-02291-2

**Published:** 2020-10-19

**Authors:** Nancy J. Sturman, Amanda Tapley, Mieke L. van Driel, Elizabeth G. Holliday, Jean I. Ball, Andrew R. Davey, Alison Fielding, Kristen FitzGerald, Neil A. Spike, Parker J. Magin

**Affiliations:** 1 Primary Care Clinical Unit, Faculty of Medicine, The University of Queensland, 8th Floor, Health Sciences Building, Royal Brisbane Hospital, Herston, Brisbane, 4006 Australia; 2 School of Public Health and Medicine, The University of Newcastle, Callaghan, NSW Australia; 3 GP Synergy, Regional Training Organisation, NSW & ACT Research and Evaluation Unit, Newcastle, NSW Australia; 4 The University of Newcastle Hunter Medical Research Institute, Clinical Research Design and Statistical Support Unit (CReDITSS), New Lambton, NSW Australia; 5 School of Medicine, University of Tasmania, Hobart, TAS Australia; 6 General Practice Training Tasmania (GPTT), Regional Training Organisation, Hobart, TAS Australia; 7 Eastern Victoria GP Training, General Practice Training Organisation, Melbourne, Australia; 8 Department of General Practice Melbourne, The University of Melbourne, Melbourne, VIC Australia

**Keywords:** Postgraduate training, Primary care education, Clinical supervision, Help-seeking, General practice training

## Abstract

**Background:**

General practice (GP) trainees may seek supervisor assistance to complete their patient consultations. This in-consultation assistance plays a key role in the supervisory oversight of trainees and in trainee learning. It may be obtained face-to-face, or using phone or messaging systems, and either in front of patients or outside their hearing. Trainee concerns about decreased patient impressions of their competence, and discomfort presenting patients within their hearing, act as barriers to seeking help during consultations. Little is known about the frequency and associations of trainee concerns about these patient-related barriers, or the various trainee-supervisor-patient configurations used to obtain in-consultation assistance.

**Methods:**

Australian GP trainees rated their frequency of use of five specific configurations for obtaining in-consultation assistance, perceived change in patient impressions of their competence after this assistance, and relative trainee comfort presenting patients outside, compared to within, patients’ hearing. Statistical analyses included descriptive statistics and multivariable logistic regression.

**Results:**

Responses were received from 778 Australian GP trainees (response rate 89%). Help-seeking configurations did not differ between trainees at different training stages, except for greater use of electronic messaging in later stages. In-consultation assistance was most commonly provided by phone between trainee and supervisor consulting rooms, or outside the trainee’s patient’s hearing. Supervisor assistance in the trainee’s room face-to-face with the patient was reported as either never or rarely obtained by 12% of respondents. More trainees (25%) perceived that patient impressions of their competence increased after help-seeking than perceived that these impressions decreased (19%). Most trainees (55%) preferred to present patients outside their hearing. Trainee age was the only variable associated with both patient-related barriers.

**Conclusion:**

Supervisors appear to have considerable influence over trainee help-seeking, including which configurations are used and trainee perceptions of patient-related barriers. In-consultation supervision may actually increase trainee perceptions of patient impressions of their competence. Many supervisors and trainees may benefit from additional educational and workplace interventions to facilitate comfortable and effective trainee help-seeking in front of patients. More work is required to understand the clinical and educational implications of different help-seeking configurations when trainees require ‘just in time’ supervisor assistance.

## Background

General practice (GP) specialist trainees (in Australia termed ‘registrars’) are expected to seek help from their GP supervisors when they lack the confidence, knowledge and/or skills to manage patient consultations independently [1]. Australian trainees complete at least three six-month terms across two different training practices, following mandatory hospital placements. They typically undertake patient consultations within the first week of their general practice terms [2, 3], with supervision available on trainee request. Training practices are accredited by regional training organisations [1], and trainees are employed by the practice [4]. Patients are not registered with particular practices or general practitioners. Help-seeking from supervisors before, during and/or after patient consultations [5] is widely believed to play a key role in patient, trainee and training practice safety [6, 7], and the professional development of the trainee [8, 9]. The particular focus of this study is help-seeking during consultations (“in-consultation help-seeking”), which affords unique opportunities. In-consultation assistance allows trainees to: maintain workflow by preventing consultation breakdowns [10]; make differential diagnoses and develop management plans without resort to temporising strategies [5]; and learn from salient, ‘just-in-time’ advice [11].

Trainees in their first 6 months of Australian GP training (“Term 1”) seek in-consultation assistance from supervisors in 11% of their consultations, and more advanced trainees seek help less frequently [9]. There are different trainee-supervisor-patient configurations for obtaining this in-consultation assistance: it may be obtained either within or outside the patient’s hearing, and either face-to-face, by phone or using internal messaging [12, 13]. Different configurations may have different implications for trainee learning [12] and patient safety [14]. Obtaining help in front of patients with the trainee, supervisor and patient all face-to-face, for example, affords learning opportunities from direct supervisor observation of trainee performance, including their interactions with the patient [15], visual inspection of the patient (e.g. diagnosis of skin conditions) and supervisor modelling of consultation and clinical skills (including skills in communicating and managing uncertainty) [6]. Opportunities are also afforded for clarification of history and negotiation of management with the patient, and confirmation of trainee findings. On the other hand, the presence of the patient may reduce the supervisor’s emphasis on teaching [12] and influence trainees to withhold sensitive information from their case presentations, differential diagnoses and management plans [16]. Currently little is known about how often the various configurations are used, and whether this changes over the course of training. This information is important in understanding the learning opportunities and clinical guidance which are provided during in-consultation supervision, and how these might be enhanced.

Different configurations may also call on different skills, and present different barriers and challenges for trainees. Assistance which is obtained in front of patients, for example, calls on the trainee to present the case within the patient’s hearing, and both trainee and supervisor need to manage patient impressions of the trainee’s competence skilfully in order to avoid the trainee losing face and the patient losing confidence in the trainee. Trainee discomfort presenting in front of patients, and trainee beliefs that patient impressions of their competence decrease when they obtain in-consultation assistance, have been reported in the literature [13]. These are likely to be barriers (referred to in this paper as “patient-related barriers”) to trainees seeking this assistance. Conducting patient-centred consultations, and maintaining patient confidence in their clinical competence, are important considerations for GP trainees [13, 17]. Trainees in hospital contexts use various strategies to present patients to senior doctors, using medically sanctioned talk to indicate areas of uncertainty [18, 19] which preserves peer and supervisor impression of trainee competence. These strategies may include casting doubt on the reliability of patient histories [18], which may be less acceptable in front of general practice patients, adding to the challenges of in-consultation help-seeking for GP trainees. In order to circumvent the risk of decreased patient or supervisor impressions of their competence, and/or reducing the patient-centredness of their consultations, trainees may leave the patient to seek in-consultation assistance, or defer their help-seeking until the patient has left [13]. It is therefore important to identify the extent of patient-related barriers, as a first step in supporting trainees to obtain appropriate in-consultation assistance.

This paper seeks to address the current gaps in our knowledge about how often the various configurations are used, and the extent of patient-related barriers to trainees obtaining supervisor assistance in front of patients. The aims of this study were 1) to investigate the specific configurations used by GP trainees for obtaining in-consultation assistance, and 2) to investigate the frequency and associations of trainees’ concerns about two patient-related barriers to in-consultation assistance (trainee perceptions of decreased patient impressions of their competence, and trainee discomfort presenting the patient within the patient’s hearing).

## Methods

### Data collection

This was a cross-sectional study conducted in 2018 within the Registrar Clinical Encounters in Clinical Training (ReCEnT) project, an ongoing multi-site cohort study described in detail elsewhere [20]. Participants were general practice trainees from three Australian states, from training organizations delivering general practice vocational training to 44% of all Australian general practice trainees [21]. These trainees complete ReCEnT data collection each 6-month training term as a part of their educational program and may also provide consent for the data to be used for research purposes. The hard-copy ReCEnT survey for this study was either completed by trainees at educational workshops or completed after workshops and returned by post.

This paper reports findings from survey items specifically concerning in-consultation help-seeking included in one round of ReCEnT data collection. The items were piloted with two trainees who did not participate in ReCEnT. Question 1 rated the frequency of use of five specific configurations for obtaining in-consultation help from supervisors, using a 5-point Likert scale. The five configurations were: 1) trainee, supervisor and patient face-to-face in the trainee’s consulting room, after supervisor interrupts their own consultation; 2) trainee, supervisor and patient face-to-face in the trainee’s consulting room, after supervisor completes their own consultation; 3) by phone between trainee and supervisor consulting rooms; 4) by phone or face-to-face outside the trainee’s patient’s hearing; and 5) using an internal electronic messaging system between trainee and supervisor rooms. These survey items are included as Additional file 1.

Question 2 rated trainee perceptions of any change in patient impressions of trainee competence after they seek in-consultation help, using a 5-point Likert scale.

Question 3 rated relative trainee comfort presenting to their supervisor outside, compared to within, the patient’s hearing, using a 5-point Likert scale.

The full survey was an iteration of a questionnaire completed six-monthly in the ongoing inception cohort study [20], which also elicited trainee and practice demographic details. See Additional file 2 for the full questionnaires used for the data collection period relevant to the current analysis.

### Analyses

Responses rating the frequency of using help-seeking configurations were dichotomised as Never/Rarely and Sometimes/Often/Always. Responses indicating perceptions of change in patient impressions of trainee competence after trainees sought in-consultation help were dichotomised as: ‘Decreases a lot’/‘Decreases somewhat’ and ‘Does not change’/‘Increases somewhat’/‘Increases a lot’. Responses rating relative trainee comfort presenting outside, compared to within, the patient’s hearing were dichotomised as: ‘Much less comfortable’/‘Somewhat less comfortable’/‘Neither more nor less comfortable’ and ‘Somewhat more comfortable’/‘Much more comfortable’. These dichotomisations were determined by a panel of medical educationalists from our author team, based on existing literature and our educational judgement that trainees who believe that patient impressions of their competence decrease, or who are more comfortable presenting outside the patient’s hearing, will be less inclined to seek in-consultation assistance in front of patients.

Analyses were programmed using STATA 13.1 and SAS V9.4. Descriptive statistics included frequencies and proportions for categorical variables and mean with SD for continuous variables. The frequencies of categorical variables were compared between outcome categories using a Chi-square test. For continuous variables, means were compared using a t-test.

The dichotomised responses to questions 2 and 3 were used as dependent variables in two logistic regression analyses, using trainee and practice demographic information as independent variables.

Univariate and multivariable logistic regression analyses were undertaken to estimate the associations between outcomes for Question 2 (perceived change in patient impressions of trainee competence) and Question 3 (relative trainee comfort presenting outside the patient’s hearing) and the independent variables.

All covariates with a *p*-value < 0.20 in the univariate analyses were considered in the multiple regression model. Once the model with all significant covariates was fitted, model reduction assessed covariates with *p* > 0.20 in the multivariable model. These were tested for removal and if removal did not substantially change the model the covariate was removed from the final model. A substantive change to the model was defined as any covariate in the model having a change in the effect size (odds ratio) of greater than 10%. *P* values < 0.05 were considered statistically significant.

In post-hoc analyses, we examined differences in the use of help-seeking configurations, and trainee responses to Questions 2 and 3, across training (Terms 1, 2 and 3) using a chi square test for trend. A sub-group of participants who responded Never/Rarely to both trainee, supervisor and patient face-to-face configurations (configurations 1 and 2) was also identified. Associations between this sub-group and responses to Questions 2 and 3 were explored using a Chi square test.

The study has ethics approval from the University of Newcastle Human Research Ethics Committee Reference H-2009-0323.

## Results

Responses were received from 778 of 876 eligible trainees (response rate 89%). See Table 1 for a summary of participant demographics.

**Table 1:** Summary of participant demographic information (*n* = 778)

Trainee Gender	female *n* = 452 (58.1%)
	male *n* = 326 (41.9%)
Trainee Australian or overseas primary medical qualification	Australian *n* = 643 (82.7%)
Trainee Term of Training [1–3]	Term 1 *n* = 482 (62.0%)
	Term 2 *n =* 77 (9.9%)
	Term 3 *n* = 219 (28.2%)
Trainee working part-time or full-time in general practice	Part-time *n* = 176 (23.1%)
	Full-time *n* = 586 (76.9%)
Trainee post-graduate medical qualifications	Yes *n* = 305 (39.5%)
Years worked as medical practitioner prior to GP training	Mean (SD) = 3.4 (3.1) Years
First term in current training practice	Yes *n* = 85 (11.1%)
Rurality of current practice (ASGC-RA) ^a^	Remote/outer regional *n* = 81 (10.4%)
	Inner regional *n* = 215 (27.7%)
	Major city *n* = 481 (61.9%)
Relative socioeconomic advantage of current practice (SEIFA index)^a^	Mean (SD) = 5.5 (2.9)
Size of current training practice^b^	Small *n* = 332 (43.8%)
	Large *n* = 426 (56.2%)

^a^Increased rurality indicated by higher ASGC-RA, and more disadvantaged locations indicated by lower SEIFA

^b^small ≤5 FTE GPs; large ≥6 FTE GPs

### Use of specific configurations for in-consultation help-seeking

The frequency of use of each strategy for obtaining in-consultation assistance from a supervisor is reported in Table 2 (See Additional file 3 for full non-dichotomised frequencies). There was no significant trend across training in the frequency of these configurations, except for electronic messaging which became more common as training progressed (although it remained the least often used strategy at all stages). The percentage of participants who reported never or rarely obtaining help face-to-face with trainee, supervisor and patient (whether or not the supervisor interrupted their own consultation) was 12.4% (15.7% for Term 3, 11.8% for Term 2, and 11.0% for Term 1, *p* value = 0.09). When this face-to-face strategy was used, supervisors were reported to more often complete their own consultations before providing assistance than to interrupt them (see Table 2).

**Table 2:** Reported use of specific configurations for in-consultation help-seeking

Strategy for obtaining in-consultation advice from supervisor(s)	Number (%) of GP trainees reporting use of this strategy sometimes, often or always				Chi-square for trend *p*-value
	Term 1	Term 2	Term3	Total	
Trainee, supervisor and patient face-to-face, after supervisor interrupts own consultation	298 (62.1%)	47 (62.7%)	136 (63.0%)	481 (62.4%)^^^	0.82
Trainee, supervisor and patient face-to-face after supervisor completes their own consultation	368 (76.7%)	61 (80.3%)	156 (71.9%)	585 (75.7%)*	0.22
By phone within the patient’s hearing	388 (80.8%)	64 (87.7%)	176 (81.1%)	628 (81.6%)^#^	0.79
By phone or face-to-face outside the patient’s hearing	363 (75.8%)	63 (84.0%)	163 (74.4%)	589 (76.2%)*	0.86
Internal electronic messaging system*	101 (21.0%)	16 (21.6%)	70 (32.0%)	187 (24.2%)*	0.002
Total	482	77	219	778	

^^^total 771; ^#^total 770; *total 773

*Electronic intra-practice messaging system, usually embedded in practice software

### Barriers to seeking help

Nineteen percent (*n* = 149, 95% CI: 16–22%) of trainees perceived that in-consultation help-seeking decreased (‘somewhat’ or ‘a lot’) patient impressions of their competence, 57% (*n* = 441, 95% CI: 53.2–60.1) perceived that patient impressions were unchanged, and 25% (*n* = 188, CI: 21.3–27.3)) perceived that in-consultation help-seeking increased patient impressions of their competence.

On multivariable analysis, trainees who felt that in-consultation help-seeking decreased patient impressions of their competence were more likely to be younger (mean age 31 years v. mean 33 years, OR 0.91 (95% CI: 0.87, 0.96) for each year of age), have post-graduate medical qualifications (OR 1.58 (95% CI: 1.07, 2.34)), or to have worked at their current training practice for less than 6 months full-time equivalent (OR 3.85, 95% CI: 1.33,11.11) (*p* < 0.001, 0.021 and 0.013 respectively) (see Table 3). On univariate analysis, trainees who felt that in-consultation help-seeking decreased patient impressions of their competence were more likely to work in a major city than inner regional or outer regional and remote areas, (OR 0.40 (95% CI: 0.25, 0.63) and 0.39 (95% CI: 0.19, 0.81), *p <* 0.001 and 0.012 respectively) and in relatively socio-economically advantaged areas (OR 1.09 (95% CI: 1.02, 1.17) for each decile of disadvantage, p 0.01), although none of these associations were statistically significant after controlling for other variables in the multivariable analysis. There were no differences between trainees at different stages of training.

**Table 3:** Factors associated with trainees indicating that patient impressions of their competence decrease after they obtain in-consultation help ^a^(total sample = 778; *n* = 712 observations used)

Factor group	Variable	Class	Univariate		Adjusted	
			OR (95% CI)	P	OR (95% CI)	p
Registrar factors	Worked at practice previously	Yes	0.34 (0.15, 0.76)	0.008	0.26 (0.09, 0.75)	0.013
	Has previous health qualification	Yes	1.57 (0.96, 2.56)	0.07	1.63 (0.91, 2.90)	0.10
	Has post-grad medical qualification	Yes	1.44 (1.00, 2.06)	0.05	1.58 (1.07, 2.34)	0.021
	Registrar age		0.92 (0.89, 0.96)	< 0.001	0.91 (0.87, 0.96)	< 0.001
	Years prior to GP training		0.92 (0.86, 0.99)	0.02	1.05 (0.96, 1.15)	0.31
	Training region	2	0.16 (0.04, 0.70)	0.015	0.22 (0.05, 1.04)	0.056
	Comparator:1	3	1.17 (0.66, 2.05)	0.59	0.69 (0.37, 1.31)	0.26
		4	1.30 (0.77, 2.20)	0.33	0.80 (0.42, 1.53)	0.50
		5	0.57 (0.29, 1.11)	0.099	0.62 (0.30, 1.28)	0.19
Practice factors	Rurality (ASGC-RA) ^b^	Inner regional	0.40 (0.25, 0.63)	< 0.001	0.64 (0.34, 1.21)	0.17
	Comparator: Major City	Outer regional remote	0.39 (0.19, 0.81)	0.012	0.87 (0.37, 2.07)	0.75
	SEIFA decile (socioeconomic index for area of disadvantage) ^b^		1.09 (1.02, 1.17)	0.010	1.03 (0.96, 1.11)	0.42

^a^Outcomes were dichotomised as ‘Decreases a lot’/‘Decreases somewhat’ and ‘Does not change’/‘Increases somewhat’/‘Increases a lot’

^b^Increased rurality indicated by higher ASGC-RA, and more disadvantaged locations indicated by lower SEIFA

Fifty-five percent (*n* = 425, 95% CI: 51.2–58.2) of trainees were more comfortable (‘somewhat’ or ‘a lot’) presenting outside the patient’s hearing, 40% (*n* = 312, 95% CI: 36.8–43.7) reported no difference in comfort level, and 5% (*n* = 40, 95% CI: 3.8–6.9) of respondents were less comfortable. Trainees who were more comfortable presenting outside the patient’s hearing were more likely to be female (OR 1.5 (95% CI: 1.10, 2.03); *p* = 0.001) and younger (mean age 32 versus 33 years, OR 0.97 (95% CI: 0.95, 1.00) for each year of age, *p* < 0.001) on multivariable analysis (see Table 4). There were no significant differences between trainees at different stages of training.

**Table 4:** Factors associated with trainees being more comfortable presenting outside (compared to within) patients’ hearing ^a^ (total sample = 778; *n* = 705 observations used)

Factor group	Variable	Class	Univariate		Adjusted	
			OR (95% CI)	p	OR (95% CI)	p
Registrar factors	Registrar gender	Female	1.46 (1.10, 1.95)	0.001	1.50 (1.10, 2.03)	0.001
	Registrar age		0.97 (0.95, 1.00)	0.02	0.97 (0.95, 1.00)	0.02
Practice factors	Practice size	Small	1.22 (0.92, 1.63)	0.17	1.25 (0.92, 1.69)	0.15
	SEIFA index		0.96 (0.91, 1.01)	0.09	0.95 (0.90, 1.00)	0.05

^a^Outcomes were dichotomised as ‘Much less comfortable’/‘Somewhat less comfortable’/ ‘Neither more nor less comfortable’ and ‘Somewhat more comfortable’/‘Much more comfortable’

There was no association between never or rarely obtaining help with trainee, supervisor and patient face-to-face (whether or not the supervisor interrupted their own consultation to respond) and either perceived patient impressions of competence (chi square *p* = 0.49) or comfort presenting within the patients’ hearing (chi square *p* = 0.33).

## Discussion

We have presented more granular information than previously available about the help-seeking configurations used by Australian GP trainees, and explored trainee concerns about two patient-related barriers to face-to-face in-consultation supervision. These configurations and concerns are likely to be relevant in many clinical training contexts, despite international differences in oversight practices in general practice training [2] and across different medical contexts [22, 23]. The COVID-19 climate has recently directed attention to alternatives to face-to-face clinical and supervisory interactions in primary care. However in-consultation or ‘just in time’ trainee help-seeking, and face-to-face supervisor assistance when appropriate, remain important aspects of clinical supervision in many clinical training contexts.

Help-seeking configurations did not differ between trainees at different stages of training, except for relatively more frequent use of electronic messaging in later stages (although this remained least often used). Twelve percent of trainees (including 11% of Term 1 trainees) reported never or rarely obtaining assistance with trainee, supervisor and patient face-to-face, whether or not their supervisor interrupted their own consultation to respond. It was surprising that these configurations differed little with stage of training: less advanced trainees would have been expected to seek more face-to-face assistance, for example. It seems likely that trainee help-seeking configurations reflect supervisor rather than trainee preferences for seeking assistance (although the preferred strategy will also depend on the clinical and educational context). The relative increase in electronic messaging may reflect the increased confidence of more advanced trainees in seeking on-screen in-consultation assistance which may be visible to patients, and less need for an immediate supervisor response.

Supervisors were more likely to complete their own consultations, than to interrupt these, before providing in-consultation face-to-face assistance. This is likely to prolong the trainee’s consultation and delay their subsequent consultations, which has been identified by trainees as a barrier to help-seeking [13]. Patient-centred supervisor strategies to safely interrupt and resume their own consultations should be identified. The impact of these interruptions on supervision quality and patient care (for both patients involved) is unknown, and should also be explored.

Trainees were more likely to perceive that patient impressions of their competence increased after help-seeking than decreased, although perceptions of decreased impressions were still reported by approximately one in five trainees. It would be valuable to identify how trainees and supervisors manage help-seeking interactions to not only preserve, but actually increase, patient impressions of trainee competence (at least as perceived by the trainee), given that help-seeking has often been considered to be a risk to trainee face and self-presentation [24, 25]. Trainees who had worked in their current practice for longer than 6 months were less likely to perceive a decrease in patient impressions of their competence. It is possible that these trainees have more secure relationships with their patients, a better understanding of the local practice norms for help-seeking (the ‘regimes of competence’ set by local communities of practice [26]), and/or more familiarity with their supervisors’ knowledge, skills and attitudes, allowing them to ‘cherry pick’ [13] those supervisors who are better able to maintain favourable patient impressions. Most trainees were more comfortable summarising the patient’s presentation outside the patient’s hearing, and very few trainees were more comfortable presenting within the patient’s hearing. It is important to gain a better understanding of the factors that are involved in this discomfort, which may include situations in which some information is sensitive or prejudicial (such as trainee suspicions of patient drug-seeking, or concerns about previous management). It is important to understand why neither patient-related barrier to face-to-face in-consultation help-seeking appeared to change through training, as trainees might be expected to become progressively more skilful and comfortable with help-seeking in the patient-centred general practice context. Supervisor responses, which are beyond the influence of trainees, may again have a key impact. Educational interventions to reduce these barriers may include raising supervisor awareness of the trainee perspective, and perhaps the use of code words to convey sensitive material.

### Strengths and limitations

Strengths of this study include the large sample size and high response rate. Our study population includes a considerable proportion of Australian GP trainees and generally reflects their demographics, although our sample includes a somewhat lower proportion of rural and Term 2 trainees [21].

A limitation is that self-report data may not capture actual practice accurately, and there may have been social desirability influences on trainee responses. The study was limited to in-consultation help-seeking from GP supervisors. We did not explore any relationship between in-consultation and pre- or post-consultation assistance, or help-seeking from sources other than GP supervisors. We did not measure actual patient impressions of trainee competence, and cannot comment on the accuracy of these trainee perceptions. We are not able to comment on any relationship between the goals of help-seeking and the use of particular configurations. We have not evaluated the outcomes of the various help-seeking configurations in terms of trainee learning or patient safety, but this would be a valuable area for future research, including direct observation of trainee consultations and in-consultation supervision.

## Conclusion

Our findings advance understanding of the important area of trainee help-seeking from clinical supervisors in workplace training. Supervisors appear to have a considerable influence over the configurations used, and trainee perceptions of patient-related barriers. Many supervisors and trainees at all levels of training, may benefit from additional training in comfortable and effective face-to-face in-consultation help-seeking interactions in clinical training contexts, which enhance rather than decrease patient impressions of trainee competence. Communication skills for presenting the appropriate information about a patient within their hearing, whether by phone or face-to-face, should also be identified and taught. In order to deliver effective and appropriate ‘just in time’ assistance and oversight in clinical training, we should also continue to develop our understanding of the educational and clinical implications of different help-seeking configurations.

## Supplementary information

**Additional file 1.** Survey items specifically concerning in-consultation help-seeking.

**Additional file 2**. Reported frequency of use of specific configurations for in-consultation help-seeking across training term.

**Additional file 3.** Full questionnaires used for the data collection period relevant to the current analysis.

## Data Availability

The datasets generated and/or analysed during the current study are not publicly available on Human Research Ethics Committee advice as some participants did not explicitly agree to their data being available.

## Abbreviations

GP: General practice
ReCEnT: Registrars Clinical Encounters in Training project

## References

[1] RACGP (2018). Training Standards: Supervision and the Practice Environment.

[2] Brown JKC, Wearne S, Snadden D, Smith M (2018). Review of Australian and International Models of GP Vocational Training and Education. Victoria: EVGPT, MCCC; 2018 August.

[3] Ingham G, Plastow K, Kippen R, White N. Tell me if there is a problem: safety in early general practice training. Educ Prim Care. 2019;30(4):212-9. 10.1080/14739879.2019.1610078 31130089

[4] General Practice Supervisors Australia (GPSA) (2020). Employment Resources. RACGP.

[5] Sagasser MH, Kramer A, Fluit C, van Weel C, van der Vleuten C. Self-entrustment: how trainees' self-regulated learning supports participation in the workplace. Adv Health Sci Educ Theory Pract. 2017;22(4):931–49. 10.1007/s10459-016-9723-4 PMC5579156 27785628

[6] Morrison J, Clement T, Nestel D, Brown J (2015). Perceptions of ad hoc supervision encounters in general practice training: a qualitative interview-based study. Aust Fam Physician.

[7] Byrnes PD, Crawford M, Wong B (2012). Are they safe in there? - patient safety and trainees in the practice. Aust Fam Physician.

[8] Wearne SM, Butler L, Jones JA (2016). Educating registrars in your practice. Aust Fam Physician.

[9] Morgan S, Wearne S, Tapley A, Henderson K, Oldmeadow C, Ball J (2015). In-consultation information and advice-seeking by Australian GP trainees from GP trainers – a cross-sectional analysis. Educ Prim Care.

[10] Smith CS, Morris M, Francovich C, Hill W, Gieselman J (2004). A qualitative study of resident learning in ambulatory clinic. The importance of exposure to 'breakdown' in settings that support effective response. Adv Health Sci Educ Theory Pract.

[11] Brandenburg DC, Ellinger AD (2003). The future: just-in-time learning expectations and potential implications for human resource development. Adv Dev Hum Resour.

[12] Brown J, Nestel D, Clement T, Goldszmidt M (2018). The supervisory encounter and the senior GP trainee: managing for, through and with. Med Educ.

[13] Sturman N, Jorm C, Parker M (2020). With a grain of salt? Supervisor credibility and other factors influencing trainee decisions to seek in-consultation assistance: a focus group study of Australian general practice trainees. BMC Fam Pract.

[14] Kennedy TJT, Lingard L, Baker GR, Kitchen L, Regehr G (2007). Clinical oversight: conceptualizing the relationship between supervision and safety. J Gen Intern Med.

[15] Billett S (2002). Toward a workplace pedagogy: guidance, participation, and engagement. Adult Educ Q.

[16] Morrison JBJ, Clement T, Nestel D (2014). Ad hoc supervisory encounters between GP-supervisors and GP-registrars: Enhancing quality and effectiveness.

[17] Ingham G (2012). Avoiding ‘consultation interruptus’ a model for the daily supervision and teaching of general practice registrars. Aust Fam Physician.

[18] Lingard L, Garwood K, Schryer CF, Spafford MM (2003). A certain art of uncertainty: case presentation and the development of professional identity. Soc Sci Med.

[19] Fox R, Albrecht GL, Fitzpatrick R, Scrimshaw S, Medical uncertainty revisited (2000). Handbook of social studies in health and medicine: London.

[20] Morgan S, Magin PJ, Henderson KM, Goode SM, Scott J, Bowe SJ (2012). Study protocol: The registrar clinical encounters in training (ReCEnT) study. BMC Fam Pract.

[21] Taylor RR, Ali, Edwards D, Clarke L (2018). Australian General Practice Training Program National Report on the 2017 National Registrar Survey (2018).

[22] Melvin et al. Tensions in Assessment: The Realities of Entrustment in Internal Medicine. Acad Med. 2020;95(4):609-15. 10.1097/ACM.0000000000002991 31567171

[23] Hatala R, Ginsburg S, Hauer K, Gingerich A (2019). Entrustment ratings in internal medicine training: capturing meaningful supervision decisions or just another rating?. J Gen Intern Med.

[24] Telio S, Ajjawi R, Regehr G (2015). The "educational alliance" as a framework for reconceptualizing feedback in medical education. Acad Med.

[25] Bamberger P (2009). Research in Personnel and Human Resources Management: Emerald Group Publishing Limited.

[26] Lave J, Wenger E (1991). Situated learning : legitimate peripheral participation / Jean Lave, Etienne Wenger.

