# Supplementary material for: Configurations for obtaining in-consultation assistance from supervisors in general practice training, and patient-related barriers to trainee help-seeking: a survey study
Source: BMC Med Educ. 2020 Oct 19;20:369. doi: 10.1186/s12909-020-02291-2 (PMC7570417; doi:10.1186/s12909-020-02291-2)
Supplement: Supplementary file 1 — Additional file 1. Survey items specifically concerning in-consultation help-seeking. [file 12909_2020_2291_MOESM1_ESM.docx]

| **Q1** When you obtain advice during the consultation from your current main GP supervisor, please rate how often you use the following methods:   \| **Methods of obtaining advice during the consultation** \| **Never** \| **Rarely** \| **Sometimes** \| **Often** \| **Always** \| \| --- \| --- \| --- \| --- \| --- \| --- \| \| 1. My supervisor interrupts his/her consultation and comes into my consulting room \| 🞏 \| 🞏 \| 🞏 \| 🞏 \| 🞏 \| \| 1. My supervisor comes into my consulting room in between his/her own consultations \| 🞏 \| 🞏 \| 🞏 \| 🞏 \| 🞏 \| \| 1. My supervisor and I talk by phone, from our own consulting rooms \| 🞏 \| 🞏 \| 🞏 \| 🞏 \| 🞏 \| \| 1. My supervisor and I talk face-to-face or by phone, out of the patient’s hearing \| 🞏 \| 🞏 \| 🞏 \| 🞏 \| 🞏 \| \| 1. My supervisor and I communicate by an electronic messaging system from our desktop computers \| 🞏 \| 🞏 \| 🞏 \| 🞏 \| 🞏 \| \| 1. Other (please specify): \|  \| \| \| \| \| \|  \| \| \| \| \| \| \| \| \| \| **Q2** When my current main GP supervisor gives me advice during the consultation, I feel the patient’s assessment of my competence: \| **Decreases a lot**  🞏 \| **Decreases somewhat**  🞏 \| **Does not change**  🞏 \| **Increases somewhat**  🞏 \| **Increases a lot**  🞏 \| \| --- \| --- \| --- \| --- \| --- \| --- \| \| \| \| \| \| \| \| \| \| **Q3** Compared to presenting to my current main GP supervisor in front of the patient, I find presenting their case **outside the patient's hearing:** \| **Much more uncomfortable xxx**  🞏 \| **Somewhat more uncomfortable**  🞏 \| **No more or less uncomfortable**  🞏 \| **Somewhat more comfortable**  🞏 \| **Much more comfortable xxx**  🞏 \| \| --- \| --- \| --- \| --- \| --- \| --- \| \| \| \| \| \| \| \| |
| --- | --- | --- | --- | --- | --- | --- | --- | --- | --- | --- | --- | --- | --- | --- | --- | --- | --- | --- | --- | --- | --- | --- | --- | --- | --- | --- | --- | --- | --- | --- | --- | --- | --- | --- | --- | --- | --- | --- | --- | --- | --- | --- | --- | --- | --- | --- | --- | --- | --- | --- | --- | --- | --- | --- | --- | --- | --- | --- | --- | --- | --- | --- | --- | --- | --- | --- | --- | --- | --- | --- | --- | --- | --- | --- | --- | --- |
