# Supplementary material for: Configurations for obtaining in-consultation assistance from supervisors in general practice training, and patient-related barriers to trainee help-seeking: a survey study
Source: BMC Med Educ. 2020 Oct 19;20:369. doi: 10.1186/s12909-020-02291-2 (PMC7570417; doi:10.1186/s12909-020-02291-2)
Supplement: Supplementary file 2 — Additional file 2. Reported frequency of use of specific configurations for in-consultation help-seeking across training term. [file 12909_2020_2291_MOESM2_ESM.zip › Supplementary File Supervisor assistance itemsR2.pdf]

“Supervisor” section

*The following items relate to **supervision** in general practice.*

19. Please indicate your level of agreement with each of the statements below by rating each item on the scale shown:

	Strongly disagree	Disagree	Agree	Strongly agree
a) My current main GP supervisor encourages me to seek advice from him or her if I am uncertain	☐	☐	☐	☐
b) I am generally satisfied with the advice I obtain from my current main GP supervisor	☐	☐	☐	☐

20. Please complete the following two sentences as to how you feel about your practice:

	Decreases a lot	Decreases somewhat	Does not change	Increases somewhat	Increases a lot
a) When my current main GP supervisor gives me advice during the consultation, I feel the patient’s assessment of my competence:	☐	☐	☐	☐	☐

	Much more uncomfortabl e	Somewhat more uncomfortabl e	No more or less uncomfortabl e	Somewhat more comfortable	Much more comfortable
b) Compared to presenting to my current main GP supervisor in front of the patient, I find presenting their case **outside the patient's hearing:**	☐	☐	☐	☐	☐

21. When you obtain advice during the consultation from your current main GP supervisor, please rate how often you use the following methods:

**Methods of obtaining advice during the consultation**	Never	Rarely	Sometimes	Often	Always
a) My supervisor interrupts his/her consultation and comes into my consulting room	☐	☐	☐	☐	☐
b) My supervisor comes into my consulting room in between his/her own consultations	☐	☐	☐	☐	☐
c) My supervisor and I talk by phone, from our own consulting rooms	☐	☐	☐	☐	☐
d) My supervisor and I talk face-to-face or by phone, out of the patient’s hearing	☐	☐	☐	☐	☐
e) My supervisor and I communicate by an electronic messaging system from our desktop computers	☐	☐	☐	☐	☐
f) Other (please specify):					
