# Supplementary material for: Configurations for obtaining in-consultation assistance from supervisors in general practice training, and patient-related barriers to trainee help-seeking: a survey study
Source: BMC Med Educ. 2020 Oct 19;20:369. doi: 10.1186/s12909-020-02291-2 (PMC7570417; doi:10.1186/s12909-020-02291-2)
Supplement: Supplementary file 3 — Additional file 3. Full questionnaires used for the data collection period relevant to the current analysis. [file 12909_2020_2291_MOESM3_ESM.docx]

**Reported frequency of use of specific configurations for in-consultation help-seeking across training term**

| **Strategy for obtaining in-consultation advice from supervisor(s)** |  | **Number** (**%) of GP trainees reporting use of this strategy** | | | |
| --- | --- | --- | --- | --- | --- |
|  |  | Term 1 | Term 2 | Term3 | Total |
| Trainee, supervisor and patient face-to-face, after supervisor interrupts own consultation | Always | 21 (4.4) | 2 (2.7) | 8 (3.7) | 31 (4.0) |
|  | Often | 93 (19.4) | 11 (14.7) | 36 (16.7) | 140(18.2) |
|  | Sometimes | 184 (38.3) | 34 (45.3) | 92 (42.6) | 310 (40.2) |
|  | Never | 124 (25.8) | 23 (30.7) | 64 (29.6) | 211 (27.4) |
|  | Rarely | 58 (12.1) | 5 (6.7) | 16 (7.4) | 79 (10.3) |
| Trainee, supervisor and patient face-to-face after supervisor completes their own consultation | Always | 28 (5.8) | 1 (1.3) | 6 (2.8) | 35 (4.5) |
|  | Often | 120 (25.0) | 25 (32.9) | 52 (24.0) | 197 (25.5) |
|  | Sometimes | 220 (45.8) | 35 (46.1) | 98 (15.2) | 353 (45.7) |
|  | Never | 89 (18.5) | 12 (15.8) | 50 (23.0) | 151 (19.5) |
|  | Rarely | 23 (4.8) | 3 (4.0) | 11 (5.1) | 37 (4.8) |
| By phone within the patient’s hearing | Always | 19 (4.0) | 2 (2.7) | 14 (6.5) | 35 (4.6) |
|  | Often | 199 (41.5) | 38 (52.1) | 77 (35.5) | 314 (40.8) |
|  | Sometimes | 170 (35.4) | 24 (32.9) | 85 (39.2) | 279 (36.2) |
|  | Never | 58 (12.1) | 5 (6.9) | 28 (12.9) | 91 (11.8) |
|  | Rarely | 34 (7.1) | 4 (5.5) | 13 (6.0) | 51 (6.6) |
| By phone or face-to-face outside the patient’s hearing | Always | 21 (4.4) | 2 (2.7) | 5 (2.3) | 28 (3.6) |
|  | Often | 129 (26.9) | 19 (25.3) | 50 (22.8) | 198 (25.6) |
|  | Sometimes | 213 (44.5) | 42 (56.0) | 108 (49.3) | 363 (47.0) |
|  | Never | 89 (18.6) | 9 (12.0) | 48 (21.9) | 146 (18.9) |
|  | Rarely | 27 (5.6) | 3 (4.0) | 8 (3.7) | 38 (4.9) |
| Internal electronic messaging system | Always | 4 (0.8) | 0 | 3 (1.4) | 7 (0.9) |
|  | Often | 43 (9.0) | 4 (5.4) | 25 (11.4) | 72 (9.3) |
|  | Sometimes | 54 (11.3) | 12 (16.2) | 42 (19.2) | 108 (14.0) |
|  | Never | 94 (19.6) | 14 (18.9) | 56 (25.6) | 164 (21.2) |
|  | Rarely | 285 (59.4) | 44 (59.5) | 93 (42.5) | 422 (54.6) |
| Total |  | 482 | 77 | 219 | 778 |
